# Computational Thinking and STEM in Agriculture Vocational Training: A Case Study in a Greek Vocational Education Institution

**DOI:** 10.3390/ejihpe11010018

**Published:** 2021-03-01

**Authors:** Eleftherios Chondrogiannis, Eleni Symeonaki, Dimitris Papachristos, Dimitrios Loukatos, Konstantinos G. Arvanitis

**Affiliations:** 1 Department of Natural Resources Management and Agricultural Engineering, Agricultural University of Athens, Iera Odos 75, 11855 Athens, Greece; elhon@aua.gr (E.C.); esimeon@uniwa.gr (E.S.); dlouka@aua.gr (D.L.); 2 Department of Industrial Design and Production Engineering, University of West Attica, Thivon 250 and P. Ralli, 12244 Egaleo, Greece; dimpap@uniwa.gr

**Keywords:** computational thinking, STEM, problem solving, agriculture education and training

## Abstract

Due to the dynamic nature of the agricultural industry, educators and their institutions face difficult challenges as they try to keep pace with future demands for knowledge and skilled workers. On the other hand, computational thinking (CT) has drawn increasing attention in the field of science, technology, engineering, and mathematics (STEM) education at present and, as advanced technologies and tools emerge, it is imperative for such innovations to be sustained with knowledge and skill among STEM educators and practitioners. The present case study aims to explore the relation between CT, STEM and agricultural education training (AET) in a Greek vocational training institute (IEK), the Agriculture IEK of Metamorfosis city (IEKMC), which is active in agriculture education. The research methodology is utilized according the positivist philosophical approach through data acquisition employing a questionnaire and the quantitative (statistical) analysis of data collected. The sample consists of IEKMC educators and students selected based on simple random sampling. Based on the participants belief that CT and STEM philosophy add value in the learning process, it focuses on the application of knowledge in the real world (students) and problem solving using new technologies (educators). Educators consider “experiments” as the most significant educational tool for problem solving in teaching practice. Students rate Greek Agriculture Education and Training (GAET) higher than educators. However, the participants evaluate GAET very low due to the lack of new innovative teaching methods being introduced. Finally, there is great interest in the implementation of CT and STEM in the European Union (EU) by students and educators.

## 1. Introduction

The introduction of the computational thinking (CT) concept has provoked much discussion about the new ability of analytical and critical thinking, while there is much research on the completion of CT as well as the epistemology of STEM (science, technology, engineering and mathematics). According to the NGSS (Next-Generation Science Standards) committee, science educators are constantly expressing concern about the lack of understanding the engineering concepts. They also believe that elements of the curriculum in science education should be changed and that these elements should be included in their training. STEM contains the science of engineering that has some peculiarities, focusing mainly on the so-called design thinking, which is also related to the dimensions of computational thinking [1].

Specifically, the teaching scenarios that attempt to integrate STEM in their methodology are inseparably connected to CT dimensions. STEM epistemology is based on the transversal interdisciplinary approach, based on which there is a connection of concepts, abilities and skills to the application of scientific processes, with the ultimate goal of solving authentic problems. CT contributes to problem solving as its dimensions are a key tool for this solution and can be applied in various scientific areas. Thus, its dimensions can be used in education, as their combination between critical thinking and pre-existing knowledge, together with the STEM methodology, lead to the solution of the problem in question. Therefore, there is a close relationship between STEM epistemology and [1,2,3,4,5].

In addition, CT can be used in all sciences to solve problems, design systems, create new knowledge and comprehend the functions as well as the limitations of computer systems. Scholars argue that CT is a key competence that learners should have in addition to the other three basic skills (reading, writing and calculations). CT includes problem solving, systems design and understanding of human behavior, based on concepts that are very important for learning while additionally including a range of mental tools that reflect the broadness of the computational science field [1].

Furthermore, Agricultural Education and Training (AET) is a constantly changing industry which, due to unpredictable climate change and the related impact on global food production, has reached a critically important stage. With population increase in the coming years, food production must significantly increase at the same time as shrinking its environmental footprint. In addition, the agriculture sector requires using the innovative technologies offered by the 4th industrial revolution (Industry 4.0). Specifically, the Industry 4.0 trend is transforming the production capabilities of all industries, including the agricultural domain. The digitalization of agriculture is based on the development and introduction of new tools and machines in production (Agriculture 4.0), such as the Internet of Things (IoT), robotics, automation, new measurement tools (i.e., Unmanned Aerial Vehicle—UAV) and artificial intelligence. This fact effected on influencing the education field. In particular, Education 4.0 is coming to fill this gap through e-learning tools, the usage of personalized learning, learning by project, etc. It is of particular research interest to make use of it in practical learning through field experience, such as internships and apprenticeships [6,7,8,9]. Therefore, there is a great need for utilization of new educational techniques and models. For example, agricultural education and training have used inherently interdisciplinary contexts and involved each of the four STEM (Science, Technology, Engineering, Mathematics) subjects [10]. The last decade or so has seen the trend of “multi-disciplinary” teaching in the disciplines of science, computational science, technology, engineering and mathematics, attributing to this integration the scientific term “STEM”. STEM is an acronym of the integration of science, technology, engineering and mathematics [11].

The main research goal in this paper is to explore the use of CT and STEM in agricultural education and training through the prospect of educators and learners. For that, in this paper, a case study is presented about the usage and evaluation of CT, problem solving and STEM in Greek Agricultural Education and Training.

Τhis paper is structured in five sections as follows. Section 2 reviews briefly the theoretical background of the theoretical background of the research (CT, STEM, AET). Additionally, the methodological framework (quantitative research) is detailed in Section 3. Then in Section 4 the findings of the research are provided while their thorough analysis is developed in Section 5. Finally, in Section 6 the principal conclusions the conclusions that emerged are introduced.

## 2. Background

### 2.1. Computational Thinking (CT)

Computational thinking (CT) is based on the work of the well-known constructionist S. Papert [12,13], as Wing [14] promoted it. However, CT has recently made a dynamic reappearance in the forefront. The concept is not new and its history in computer science is quite long. Known since the 1950s and 1960s as “algorithmic thinking”, it can be argued that it is a thinking orientation based on which problems are formulated as input-to-output con-versions and algorithms are sought to implement these transformations [15].

Specifically, as in [14], CT is “solving problems, designing systems, and understanding human behavior, by drawing on the concepts fundamental to computer science”. Moreover, Cuny et al. [16], define CT as “the thought processes involved in formulating problems and their solutions so that the solutions are represented in a form that can be effectively carried out by an information-processing agent”. As Tang [17] has pointed out CT is grounded on concepts fundamental to computer science, a field that has importantly impacted society, but it is integral to modern research and the problem-solving work of STEM. Thus, CT should be embedded in the educational system as a basic learning goal to prepare students with competency in their future life [18,19,20].

The modern CT approach recognizes that it mainly concerns a mindset, rather than a distinct way of reasoning in terms of cognitive science (such as logical thinking, spatial thinking, creative thinking, etc.) consisting of a working method strongly required in computing science. The scientific community recognizes at present that CT is “a multidimensional concept that includes, as individual components, important concepts, methods and practices used by IT scientists to solve computational problems arising in various scientific fields or in everyday life” [21,22].

**Figure 1:**
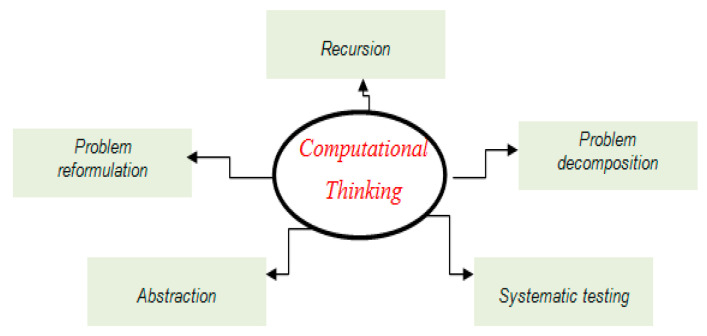
The main dimensions of computational thinking (CT).

As in [14] CT includes five (5) dimensions which are depicted in (Figure 1):

- Problem reformulation—reframe a problem into a solvable and familiar one.
- Recursion—construct a system incrementally based on preceding information.
- Problem decomposition—break the problem down into manageable units.
- Abstraction—model the core aspects of complex problems or systems.
- Systematic testing—take purposeful actions to derive solutions.

Fessakis et al. attempted as in [21] a combination of the dimensions proposed by the various definitions concluding that CT includes: creative problem solving, algorithmic problem-solving approach, solution portability, logical interference, subtraction, generalization, representation and data organization, systemic thinking, evaluation, and social impact of computing. In the same work, the dimensions of the CT included in various initiatives for the integration of CT in education that are already implemented worldwide (for example, Teaching London Computing, Computing at School [23]), were gathered and their unification was attempted.

In addition to the theoretical research, many empirical studies have attempted to integrate CT in basic education (primary, secondary). For example, Malyn-Smith and Lee [24], have facilitated the exploration of CT as a foundational skill for STEM professionals as well as the way that professionals engaged CT in routine work and problem solving. Finally, scholars and researchers have developed CT interventions on various subject domains, such as science (e.g., physics, biology), journalism and expository writing etc. [17].

Starting from the perceptions that teachers have about computational thinking and its integration in Greek schools, it is worth mentioning the research of Fessakis and Prantsoudi [22]. That specific research was conducted between IT teachers of primary and secondary education, and resulted to the outcome that IT teachers perceive computational thinking as a problem-solving type and often identify it with the algorithmic thinking dimension. Also, some of them attach equal importance to all its dimensions, while others consider that some dimensions are more important. In addition, the majority have a positive attitude towards the integration of computational thinking at all levels of education through STEM activities and interdisciplinary approaches. In addition, several studies have shown that there is a lack of training in CT issues by teachers and therefore not possible to use it in teaching practice [25,26,27,28]. Finally, research on what is considered CT in a school lesson, showed that various attempts are made by teachers, who realize how important it is to cultivate computational thinking in students for their future development, but themselves (teachers) have not received any comprehensive training on the subject. The same goes for managers, as they also do not have a complete view of what computational thinking is. However, the positive finding is that its value is recognized as a future student resource [29].

For students, the use of CT in teaching practice is positive. In a study by Adler and Kim [30], they reshaped the structure of science lessons through lesson plans using programming and simulations. The findings of this intervention showed that the students had a sufficient understanding of the physical meanings of the activities and they, in turn, would like to include similar computational thinking activities in their future classes, as long as they work as teachers.

Another study showed that the most effective time for teachers to assimilate the concepts of CT is during their studies. In particular, the ideal fields for integrating this knowledge are the educational technology courses that future teachers take, but it is necessary for the pedagogical departments to work with computer schools in order to jointly formulate an effective teaching plan for the students [31].

Regarding CT and problem solving, there is some confusion between the two concepts, as one is considered to be identical with or overlapped by the other [32]. According to Kovács and Harangus [33] the definition of problem solving is: “Problem solving is an individual’s capacity to use cognitive processes to confront and resolve real, cross-disciplinary situations where the solution path is not immediately obvious and where the literacy domains or curricula areas that might be applicable are not within a single domain of mathematics, science or reading”.

The reason for the confusion why this happens is that the strategies followed by problem solving largely match the corresponding dimensions found in CT. Research has shown that these are two independent concepts, which cannot be merged and must be evaluated separately. On the other hand, research has argued that computational thinking can be considered as a form of problem solving in a technological environment [34]. However, the view that defines computational thinking and problem solving as two different variables is confirmed by various researches and they examine their research questions on this basis. Indicatively, we refer to the research of Wong & Cheung [35] and Moon et al. [36], which support the positive effects that programming activities have on the improvement of computational thinking and study whether these activities can also benefit the development of other, general skills, including the problem-solving.

### 2.2. Science, Technology, Engineering and Mathematics (STEM) Education

STEM education is promoted in several countries due to the preparation of their citizens to understand this concept and obtain multidimensional capabilities from it to be used in modern life. Specifically, STEM helps to solve problems of real world. As a result, the curricula have been changed to include computational thinking activities from primary to high school and international teachers’ associations referring to the need to introduce STEM and computational thinking in education [37,38,39].

STEM pedagogy is based on “integrated” curricula that combine the engineering design as well as the inquiry-based teaching and learning approach. STEM is often expected to solve the low scores problem on international assessments such as the Programme for International Student Assessment (PISA) test, and the decreasing number of students who want to obtain a job related to science and technology. For example, the USA has a national plan for increasing the number of graduates with STEM degrees (i.e., engineering diplomas) to maintain America’s competitive position in the international economy [37,38,39].

**Figure 2:**
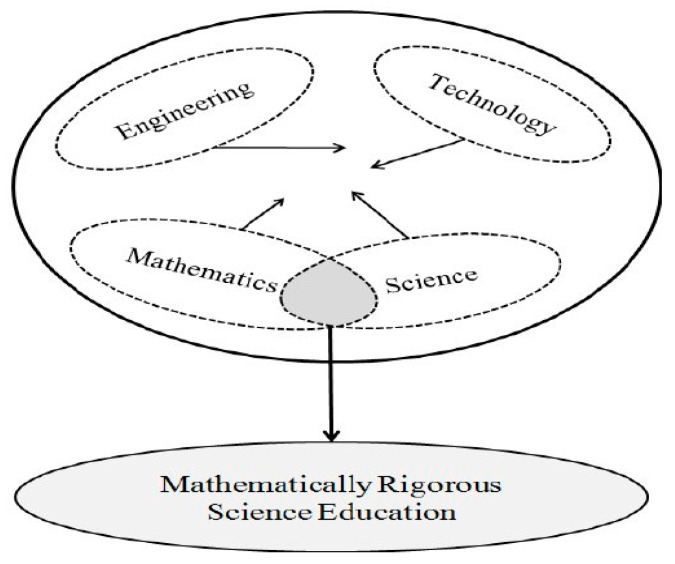
Science, technology, engineering and mathematics (STEM) education model with a particular focus on mathematics and science.

STEM as education includes the knowledge, skills and beliefs that are collaboratively constructed at the intersection of more than one STEM subject area [31]. For example, the educational model depicted in Figure 2 summarizes the scientific fields in STEM education and links (integrated) STEM education to integrated teaching at the K-12 level. According to Corlu et al. (2014), while the oval STEM shapes indicate the preservation of unique characteristics within each STEM area, such as in-depth knowledge, skills, and beliefs, the arrows from the shapes represent the teacher and student-driven interactions. The interactions exist because they are often integral rather than optional parts of the STEM areas. In addition, this educational model hypothesizes that it takes a well-educated teacher with a strong background to such interactions actually occur in the classroom settings. In terms of background, this is related to very good professional and scientific knowledge about the fields of STEM, and in addition, to satisfactory pedagogical training of the teacher. The model is designed with the potential to address all other interactions between STEM subjects [40,41]. Also, the proposition that posits mathematics is abstract but science is concrete is not supported in educational practice. This is in contrast to one view, which argues that mathematics and science are epistemologically too different to be integrated [42], but [40] believes that both subjects are related to life and are dependent on each other to construct new knowledge [40,43,44,45,46].

Many countries, including global economic powers such as the United States (USA) and the European Union (EU) are transforming their educational systems to be competitive in the age of innovation by using STEM education [47]. It is at the core of both American and EU research-based innovation strategies [31]. Specifically, in Greece, the education system is structured based on a centralized design followed by all schools, leaving little autonomy in the school units. The curricula are common to all schools and are taught by specific textbooks [48]. In the last quarter of 2017, the Educational Policy Institute (IEP) as national coordinator for Greece of the European Project H2020: “Open Schools for Open Societies (OSOS)”, advanced a call for school units to participate in the pilot phase of the OSOS project which has been implemented since the 2017–2018 school year. The focus of the project is on STEM approaches in topics that enhance both the connection of the (natural sciences) with technology, mathematics and engineering, as well as the connection with modern social concerns [49]. Various surveys [50,51,52] on STEM approaches to formal and non-formal education in Greece have shown that positive results of these approaches, such as promoting and improving the educational process enhances student performance in coding, and most teachers and students view positively the prospect of integrating STEM methodology into teaching. All of these studies indicate positive dynamics for the introduction of STEM activities in the educational process in Greece. However, the integration of more advanced technologies and digital skills remains low. The number of people with at least a basic level of digital skills remains well below the EU average, and Greece still has the lowest percentage (1.4%) of information and communication technology (ICT) experts in the EU (3.7%). On the other hand, the corresponding ratio for the number of sciences, technology, engineering and mathematics (STEM) graduates per 1000 people aged 20–29 (16.2%) is closer to the EU average (19.1%), bringing the country to 18th place. In addition, Greece ranks 6th among OECD countries in terms of the percentage of STEM graduates (26%) [53].

### 2.3. Agriculture Education and Training (AET)

Agricultural Education and Training (AET) aims to contribute to the development of the agricultural sector and rural space. The concept of rural space is identified with: (a) the natural habitat and (b) the residential rural area. Production in agriculture and the solution of its problems cannot be carried out merely with financial support or with the introduction of capital material. It is necessary to support it with: (i) school-based agricultural education, (ii) training and (iii) agriculture consulting. Specifically, in agricultural areas, the educational needs are not static, as they differ according to the peculiarities of the rural areas and the needs of the population that makes them up. Based on the above, these forms of AET are distinguished [54,55,56]:

– School-based agricultural education.
– Vocational training in agricultural professions.
– Education in rural household issues.
– Education in environmental protection issues.
– Education in agro-tourism issues.
– Education in new technologies (Agriculture 4.0).

Due to the dynamic nature of the agricultural industry, educators and their institutions face difficult challenges as they try to keep pace with future demands for knowledge and skilled workers. In order for the agriculture and agribusiness sectors to meet the challenges of sustainable food production, there is a need to view agriculture as a knowledge industry, one that requires “people of an especially high standard of education and training who can manage not only the basics of production, but also sophisticated technologies, the agro-ecological environment, the sociology and economics of their business” [56,57].

### 2.4. AET, CT and STEM

The development of 21st-century skills by students has recently been raised as an essential issue in the scope of education. Such skills include a wide range of knowledge, work habits and character traits which are expected to be applied in all academic or professional subject areas as they are claimed to be critically important for success in today’s world. Hence, CT is considered to be “one of the 21st-century skills and correlatively becomes ever more vital in today’s increasingly technological world” (i.e., Agriculture 4.0) [58].

There is also wide acknowledgement that CT methods are critical to—and sometimes transformative in—a variety of STEM fields, thus it is sought to integrate skills from CT to STEM. For example, a maths teacher could refer to the chi-square testing distribution (χ2) and then have students examine a company’s seed production to check if this distribution is verified [59].

Agricultural careers of the future will require more knowledge and skills related to science, technology, engineering, and mathematics (STEM). STEM will be important for ensuring an adequate food supply, economic well-being, safety, new industries, and an improved standard of living in developing countries. Up to the present, agricultural education has used inherently interdisciplinary contexts and involved each of the four STEM fields. According to the Association of Public and Land-grant Universities (APLU) and the USA National Research Council [60,61], STEM can assist in addressing the stagnation of student achievement in this concept.

Therefore, AET should help in the creation of a 21st-century workforce able to address social, economic, and environmental challenges through the STEM educational concept (model). The USA National Research Council [61] went as far as suggesting the STEM term should be modified (by adding agriculture as a new parameter) to science, technology, engineering, agriculture, and mathematics (STEAM). Despite the calls for increasing integration of STEM into agricultural curricula, a research gap has made it difficult to address it through policy and teacher preparation. In addition, the evolution of STEM in agricultural education has prompted researchers to explore the attitudes and perceptions of students toward this concept [62,63].

Finally, a key part of the STEM education philosophy, much like agricultural education, has stressed the importance of connecting content knowledge, STEM knowledge, real-world issues, and problem-solving skills [64]. According to [65], the teaching of STEM needs to engage the students in “motivational activities that integrate the curriculum to promote hands-on and other related experiences that would be needed to help solve problems as they relate to their environments” [65]. In addition, school-based agricultural education (SBAE) has been so diverse that the philosophy of AET has emphasized the process of learning by covering the specific content learned [64,66].

## 3. Research Methodology

### 3.1. Method

The present survey (case study) aims to explore the relation between computational thinking, STEM and agriculture education and training in a Greek vocational training institute (IEK) as it has important value nowadays, especially in the agriculture sector of an economy. In particular, teachers’ and students’ views on CT and STEM are recorded and analyzed. This is approached according to the positivist philosophical method through the collection data with the help of a questionnaire and the quantitative (statistical) analysis of research data (quantitative methodology). In addition, the research follows the descriptive method since, it deals with the collection of data at a specific time, in order to analyze social events, situations and phenomena or determine the relationships that exist between them (CT, STEM, AET) [64,66].

### 3.2. Framework and Research Questions

**Figure 3:**
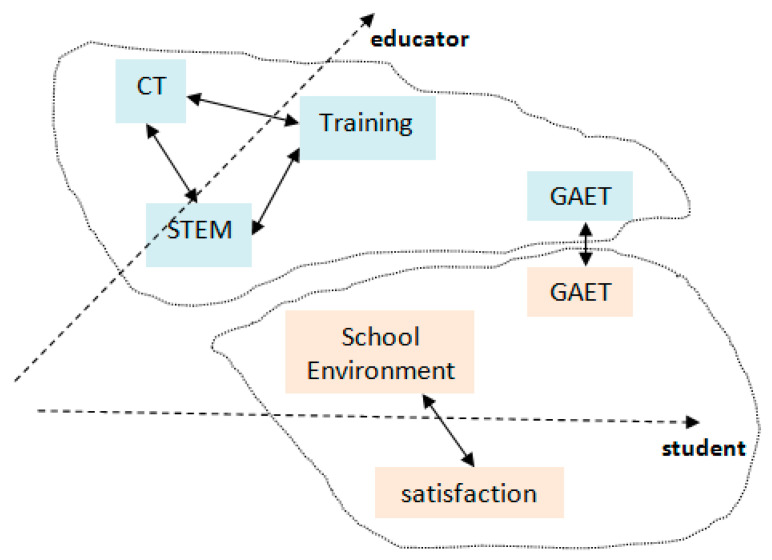
Research framework.

We propose a research framework (according to main research goal) with two (2) axes (educators, students) which interact between them as follows (Figure 3):

- 1st axis: this involves the opinion of CT, opinion of STEM philosophy, educator’s training and evaluation of Greek agriculture education and training (GAET).
- 2nd axis: this involves opinion of school environment, satisfaction of school environment and evaluation of GAET.

**Figure 4:**
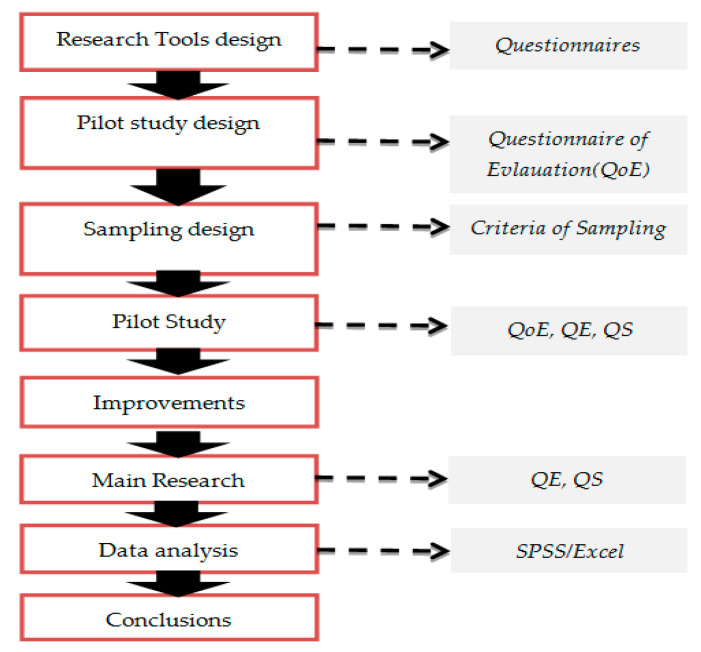
Steps of research procedure.

According to the theoretical review and research framework (as depicted in Figure 4) the research questions (RQ) are structured as follows:

- RQ1: Is there relation between opinion of STEM philosophy and opinion for CT?
- RQ2: Is there relation between educators’ training and opinion for CT?
- RQ3: Is there relation between opinion of STEM philosophy and Educators’ training?
- RQ4: Is there relation between opinion for school environment and Satisfaction of School Environment?
- RQ5: IS there relation between GAET (educators’ side) and GAET (students’ side)?

### 3.3. Participants

This survey (case study) focuses on the relation between CT and AET in a Greek vocational training institute (IEK) that took place in the Agriculture IEK of Metamorfosis city (IEKMC) in Attica. This IEK offers 11 professional specialties in the fields of Health, management, agriculture and security. It has 5 permanent staff and more than 70 temporary staff. It participates in European programs (i.e., Erasmus ++), while it has its own laboratory infrastructure that covers all specialties. It belongs to level 5 of the European Qualification Framework. It grants a National Diploma of Professional Specialty, Education and Training, level 5, after certification exams per professional specialty [67].

Specifically, IEKMC offers vocational training in viticulture and oenology. The wine sector is one of the most significant sectors of Greek viticulture and agricultural production in general. The “Viticulture and Oenology Technician” is an employee occupied in viticultural, winemaking or mixed wine companies of various forms with the scope of cultivating vineyards and producing wine. The population of this survey is shown in Table 1.

### 3.4. Research Procedure

The research process includes the following steps (Figure 4):

- Research tools design (questionnaire of educators, QE, questionnaire of students, QS) based research framework;
- Pilot study design;
- Sampling design;
- Pilot study;
- Improvements to research tools;
- Main research;
- Data analysis (statistical analysis);
- Conclusions.

The pilot research includes the following steps:

- Finding respondents (e-mail);
- Informing participants about the pilot research;
- Issuance of a survey questionnaire and main assessment questionnaire (pilot research);
- Collection of questionnaires;
- Data processing/conclusions and improvements.
- The main research includes the following steps:
- Finding respondents (e-mail);
- Informing participants about the main research;
- Issuance of the main research questionnaire;
- Collection of questionnaires;
- Data processing/conclusions and improvements.

### 3.5. Tools

**Figure 5:**
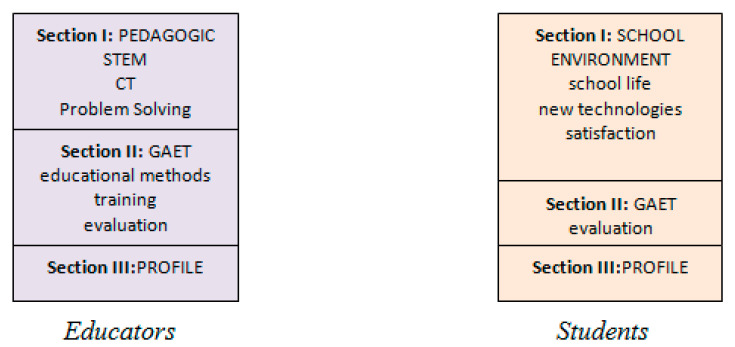
Structure of tools (questionnaire of educators, QE, questionnaire of students, QS).

In this research two special questionnaires (tools) were designed based on the research framework and theoretical review as depicted in Figure 5. The questions of the research questionnaire are divided in three types: dichotomous, multiple options and Lickert scales. In particular, the structure of the research questionnaires is set as forth below:

- Questionnaire of Educators (QE)
  - Section 1 (Pedagogic). It includes three basic questions with 12 items (totally) about STEM philosophy, CT and problem-solving methodology.
  - Section 2 (GAET). It includes three basic questions with 9 items (totally) relative to the usage of CT, STEM and problem-solving methodology in GAET, educators’ training and overall evaluation of GAET.
  - Section 3 (Profile). It includes socio-demographic characteristics (gender, education, work experience, employment situation).
- Questionnaire of Students (QS)
  - Section 1 (School environment). It includes three basic questions with 12 items (totally) about school life, using of new technologies and overall satisfaction of school environment (IEK).
  - Section 2 (GAET). It includes questions about overall evaluation of GAET.
  - Section 3 (Profile). It includes socio-demographic characteristics (gender, age).

The completion of these questionnaires (QE, QS) is anonymously performed and the answers are confidential. Regarding the pilot study’s questionnaire (questionnaire of evaluation, QoE), this is used to evaluate the research tools of the main research. This questionnaire is also completed anonymously and the answers are confidential. 8 people (4 instructors, 4 students) agreed to answer the pilot study questionnaire. The results of the pilot research help to improve the QE and QS (recording comments-comments for improvement by the respondents), while they are also a tool for validation certification (face validity). Phenomenal validity is an elementary form of validity, and shows that a measurement tool (questionnaire) seems to appreciate the concept that is said to count. In general, this type of validity facilitates simple surveys (e.g., small sample size), such as the present, because it avoids the difficult process of structural validity, which requires a large sample size and weighted scales, etc. [68,69].

In particular, the QoE evaluation criteria concern:

- the format of the research questionnaire (legible letters, font, color, easy-to-use format, etc.);
- the structure of the research questionnaire (series of questions, thematic units);
- the content of the research questionnaire (questions, explanations);
- the satisfaction of the research goal (main research goal);
- the overall satisfaction by the research tools.

The Lickert scale (5 points) is used to investigate the evaluation criteria (excluding Individual Profile). The questions are closed type, except for question 2. The types of question used are similar (dichotomous, multiple choice) to the questionnaires of the main survey.

### 3.6. Sampling

In the present work the sample selection was done with the help of the avalanche method which belongs to the non-probability sampling methodology. In avalanche sampling, the individuals selected have characteristics that are of interest to the research, which in turn bring the researcher into contact with other individuals with similar characteristics [68,69].

The research population includes all teachers and students of the specialty “Viticulture and Oenology Technician” of IEKMC. The main control characteristic used to participate in the sample is to work (teacher) or study (student) in the specialty “Viticulture and Oenology Technician” during the research period. Any demographic differences that may exist, compared to a probability sample, do not appear to create a discriminatory attitude, as it concerns a case study with a very specific population [70]. Also, despite the problems that may arise from the possibility of the same dating network participating in the sample, this is minimized as the sample size increases. For this reason, the sample sizes in the present study exceed 50% of the specialty population. The survey took place during the autumn of 2020.

In the present research, the questionnaire was disseminated between a group of educators and students by promoting additional respondents through an informal online network, using the “word of mouth” technique. The sample was selected according to the following criteria (Κ_i_):

- K_1_: teacher or student of the ΙΕΚΜC specialty “Viticulture and Oenology Technician”.
- K_2_: the ease of accessing the respondent,
- K_3_: free choice of answering the questionnaire or not.

The sample consists of 22 educators (QE) and 24 students (QS), all of whom completed the questionnaires.

### 3.7. Pilot Study

The pilot study concerns the testing and testing of the main research questionnaires (QE, QS) in a small sample of 8 people (4 students, 4 teachers) belonging to the IEKMC educational community and the specialty “Viticulture and Oenology Technician”. It took place in September 2020 and the avalanche method was used. QoE was used with the following results:

- Questionnaire format (QE, QS): significant satisfaction (75%—very satisfied) was found from the questionnaire format (QS) for students and, respectively, significant satisfaction (75%—sufficient satisfaction) from the questionnaire format (QE) for teachers.
- Questionnaire structure (QE, QS): very significant satisfaction (100%—very satisfied and sufficient satisfaction) was found from the structure of the questionnaire (series of questions, topics) for students (QS) and respectively, significant satisfaction (75%—sufficient satisfaction) in the form of a questionnaire (QE) for teachers.
- Questionnaire content (QE, QS): significant satisfaction (75%—very satisfied and sufficient satisfaction) was found from the content (questions, explanations) for the students (QS) and respectively, significant satisfaction (75%—very satisfied) from the format of the questionnaire (QE) for teachers.
- Satisfaction of the research goal (main research goal): it was observed from the results of the pilot research, that there is significant satisfaction (75%—very satisfied and sufficient satisfaction) of the content (questions, explanations) for the students (QS) and respectively, significant satisfaction (100%—very satisfied) from the form of the questionnaire (QE) for teachers.
- Overall questionnaire satisfaction: observed from the results of the pilot survey, very significant satisfaction (100%—very satisfied and sufficient satisfaction) from the structure of the questionnaire (series of questions, topics) for students (QS) and respectively, significant satisfaction (75%—very satisfied and sufficient satisfaction) from the form of the questionnaire (QE) for teachers.
- Verbal performance problems: there were few observations for verbal performance of the questions (grammar-syntax errors), 1 and 2 of the QE questionnaire and questions 3 and 4 of the QE questionnaire respectively.

Finally, there were no further comments on the main survey questionnaires. Overall, the observations helped in their final design (improvements).

### 3.8. Validation

The validity of the questionnaires (QE, QS) showed the following:

- content validity: a research questionnaire should fully cover the dimensions of the main research objective [68] so it was considered appropriate, the design of QS and QE was based on the research framework developed in the context of this research, and it examined in detail two key research factors (CT, STEM).
- criterion validity: this became impossible because the use of a criterion, i.e., an existing reliable and valid questionnaire (weighted), which concerns the investigation of the main research purpose for GAET, was not found. Here, perhaps, there was a gap in terms of researching CT and STEM views on AET.
- apparent validity: the results of the pilot survey showed significant satisfaction since in all indicators (≥50%) for both questionnaires. It was obvious that the apparent validity is a primary approach to the validity of the questionnaires, due to its subjective dimension [68,69].

### 3.9. Data Analysis

The statistical techniques used aimed for: (i) descriptive presentation of the sample for better presentation of the research sample and understanding of its aspects; and (ii) correlations in order to draw conclusions. Also, the corresponding factors were designed for all complex variables (a. Use the total sum of the answers to the questions that make up each factor, b. Analyze → descriptive statistics → frequencies [SPSS]) such as:

STEM Philosophy (STEM): the total score was calculated empirically as follows:

STEM = [∑choice of item response_i_]/n (1)

for i = {1...5}, n = 5 (items 1–5/Section Ι–question 1/Educator tool).

Computational Thinking (CT): the total score was calculated empirically as follows:

CT = [∑choice of item response_i_]/n (2)

for i = {1...4}, n = 4 (items 1–4/Section Ι–question 2/Educator tool).

Educator’s Training (ET): the total score was calculated empirically as follows:

ET = [∑choice of item response_i_]/n (3)

for i = {1...3}, n = 3 (items 1–3/Section Ι–question 5/Educator tool).

GAET educator side (GAET-E): the total score was calculated empirically as follows:

GAET-E = [∑choice of item response_i_]/n (4)

for i = {1...3}, n = 3 (items 1–3/Section Ι–question 6/Educator tool).

School Environment (SE): the total score was calculated empirically as follows:

SE = [∑choice of item response_i_]/n (5)

for i = {1...7}, n = 7 (items 1–7/Section Ι–question 1).

Satisfaction (SAT): the total score was calculated empirically as follows:

SAT = [∑choice of item response_i_]/n (6)

for i = {1...4}, n = 4 (items 1–4/Section Ι–question 2).

The responders’ answers were noted anonymously they were coded and entered into SPSS Statistics version 21, in order to achieve the interpretation of results and reach conclusions.

## 4. Results

### 4.1. Educator Survey

**Figure 6:**
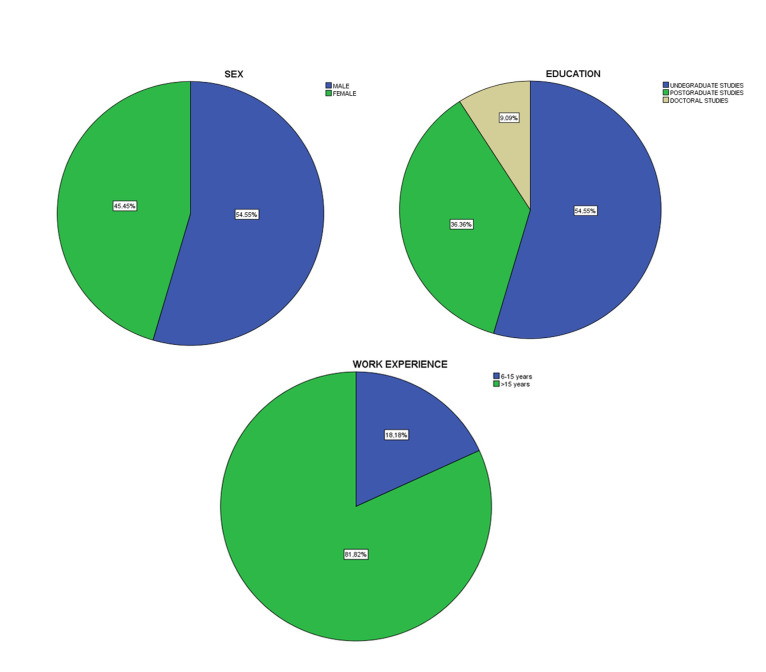
Profile of demographic variables: sex, level of studies and work experience.

In the sample of the case study, the percentage of male participants (54.55%, 12 people) was slightly higher than this of females’ participants (45.45%, 10 people), while in the level of studies, the undergraduate participants accounted for a large percentage (54.55%, 12 people) followed by the postgraduate participants (36.36%, 8 people) and Ph.D. graduates (9.1%, 2 people). Regarding work experience, the majority of the sample belonged to the upper periods (>15 years) with 81.8% (18 people) (Figure 6).

In the part of questionnaire about the opinion of STEM philosophy, all respondents agreed that from ‘often’ to ‘always’ (responds) for STEM (see Table 2): (a) it emphasizes the application of knowledge to real-world problems, (b) it emphasizes the critical thinking, (c) it supports the problem solving (d) it enhances the creativity and (e) it is important for economic development.

Table 3 summarizes, as follows, the opinion of CT, where some deductions can be observed as below:

- The most important opinions expressed were the following: (a) it helps in the problem solution (average 3.55) and (b) it is useful for the utilization of Information Technology and Science (average 3.55).
- the least important opinion was linked to STEM education (average 2.09).

Respectively, the opinion of the didactic methodology of problem solving, where the following can be seen (see Table 4):

- The most important opinions were the following: through the classical experiment (average 3.91).
- The least important opinions were the following: simulation (average 3.09) and theoretical analysis (average 3.09).

The part of the questionnaire concerning the opinion of the utilization of curriculum and infrastructure in GAET, indicated the dissatisfaction of the survey participants (average ≤ 2.0), while the part of questionnaire about the opinion of the educator’s training, indicated their dissatisfaction (average ≤ 2.0) as well. Overall dissatisfaction (average of ≤2.0) was additionally expressed for GAET. The overall evaluation is concerning GAET with regard to the utilization of CT, STEM and problem solving in the teaching processes. Finally, the correlation analysis between opinion of STEM and opinion of CT was checked with the aid of the Spearman’s rho correlation coefficient, resulting in an important positive correlation with 0.728 (correlation coefficient) and a high significant level of 0.000 (Sig. < 0.01) as presented in Table 5:

### 4.2. Student Survey

**Figure 7:**
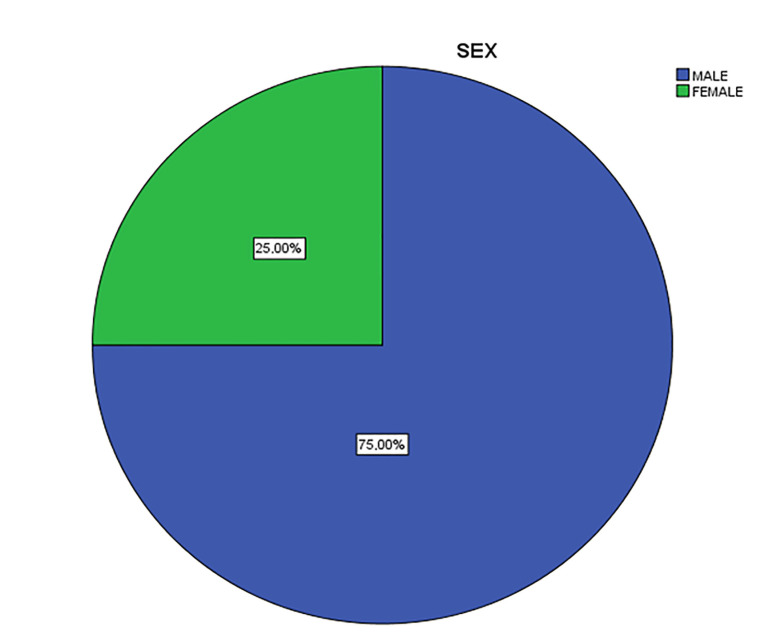
Profile of sex.

In the sample of the case study, the percentage of male students (75%, 18 people) was higher than female students (25%, 6 people) as depicted in Figure 7. Regarding age, all respondents of the sample belonged to the older age periods (>24 years).

In Table 6 the opinion of the school environment (SE) is summarized and the following deductions can be obtained:

- The most significant t opinions were those regarding: (a) the application of knowledge to real world problems (average 4.75) and (b) the use of models or prototypes in problem solving at school or life (average 3.75).
- The least important opinions were the following: using algorithms for problem solving (average 0.88) and I can solve problems more easily (average 1.13).

In the part of the questionnaire regarding the evaluation of the exploitation of the new technologies (information technology, internet etc.) in IEKMC, the results indicate that the majority of the sample agrees that there is utilization of new technologies (75%, 18 people). On the other hand, the overall satisfaction of IEKMC (SAT) results in dissatisfaction with all factors (Table 7).

**Figure 8:**
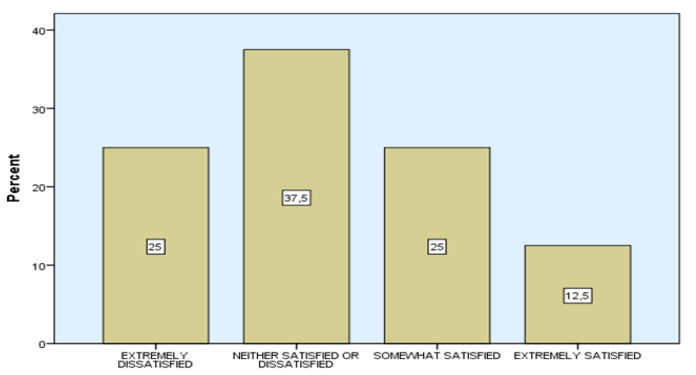
The profile of responses of SAT (overall satisfaction with school environment at IEKMC).

In addition, concerning the overall satisfaction of GAET, the results indicate medium satisfaction (average 3.0) as depicted in Figure 8. Finally, the correlation analysis between SE and SAT was checked with the aid of the Spearman’s rho correlation coefficient, resulting in a medium important positive correlation with 0.478 (correlation coefficient) and a significant level of 0.018 (Sig. < 0.05) as presented in Table 8.

### 4.3. Evaluation Comparison of Greek Agriculture Education and Training (GAET) between Educators and Students

**Figure 9:**
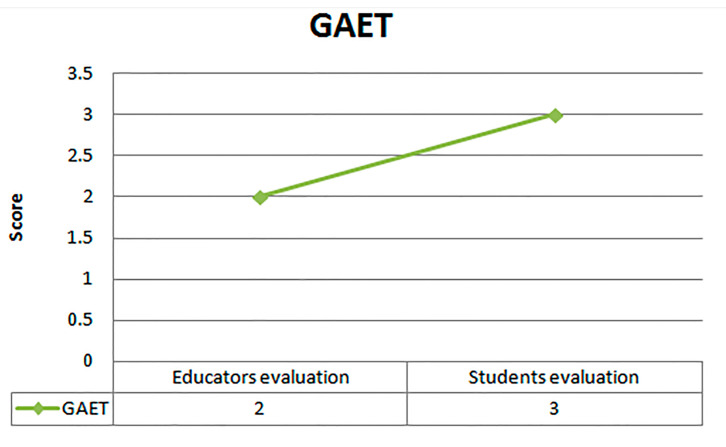
Comparison between educators and students evaluation of Greek agriculture education and training (GAET).

The comparison between the educators’ and students’ evaluations of GAET shows that the students evaluate the GAET higher (3.0) than the educators (2.0) as presented in the diagram of Figure 9.

## 5. Discussion

In the present research STEM philosophy is considered to have a very powerful relation with CT according to the responses of the participants (educators). This indicates that the STEM is inextricably linked to the CT dimensions. The STEM framework is based on the connection of concepts, skills and abilities and the use of scientific processes, with the ultimate goal of problem solving. CT contributes to this problem solving as its dimensions are a key tool for this solution and can be applied in various scientific areas [1,2,3,4,5].

Correspondingly, there is a relatively medium relationship between the view of the school environment and its evaluation by the research respondents. No relationship whatsoever was found between educator’s training and STEM philosophy, nor between educator’s training and CT in accordance with the participants’ responses in the present study.

In literature, CT involves solving problems, designing systems, and understanding human behavior, by drawing on the concepts fundamental to computer science. In addition, CT is then considered one of the 21st-century skills, especially in Agriculture 4.0. digitalization (i.e., IoT, robotics, artificial intelligence (AI) etc.), the socio-technical process of applying digital innovations, is an increasingly ubiquitous trend. In agriculture it is thus expected to provide technical optimization of agricultural production systems, value chains and food systems [58,59,71].

Educators consider STEM education important because it promotes critical thinking, helps in problem solving and applies knowledge in the real world, while also contributing to the country’s economic development. On the other hand, they consider important for CT, the fact that it assists in problem solving and the usage of new technologies, yet they find its connection with STEM education and philosophy to be of low value. In relevant research, teachers realize how important the cultivation of CT in students is for their future development, and recognize its value as a future resource for students [29].

Furthermore, the educators consider the teaching practice of experimental conduct as the most significant educational tool for problem solving, while they consider that there is no utilization in the Greek educational system of CT, STEM and didactics for problem solving. They generally evaluate GAET very low due to the lack of introduction of new innovative teaching methods. This is confirmed by the Organisation for Economic Cooperation and Development (OECD) statistics, where Greece, the integration of more advanced technologies and digital skills remain low. The number of people with at least a basic level of digital skills remains well below the EU average [53].

It is imperative to develop the CT skill set among STEM educators and students to sustain scientific revolution. Also, CT enables individuals to become more efficient problem solvers by teaching them to recognize computable problems and approach the problem-solving process skillfully [14,17,72]. Moreover, agricultural professions of the future are expected to require more knowledge and skills related to science, technology, engineering, and mathematics, such as STEM. In literature, the interconnection of STEM and AET offers significant possibilities for the development of problem-solving skills [60,61].

When teaching STEM, as Ejiwale noted in [64], the special importance of engaging students in “motivational activities that integrate the curriculum to promote hands-on and other related experiences that would be needed to help solve problems as they relate to their environments”.

In addition, student participants consider the application of knowledge in the real world to be the most important, while the use of algorithms to solve problems the least important. On the other hand, students’ rate GAET higher than educators. This indicates the need to introduce CT, problem-solving methodology and STEM into GAET. Drawing upon the growing interest in integrating CT in STEM education, the field dedicated many efforts to promote and examine students’ CT skills. Many researchers have illustrated the importance of teaching and learning CT and its integration in other subject domains [17].

Regarding the Greek educational system, the research findings showed that there is no utilization of new and innovative educational methods such as CT and STEM, while it lags behind in the use of new technologies (information and communication technologies). This may be because the education system is structured based on a centralized design followed by all schools, leaving little autonomy in the school units [48]. However, recent research in Greece shows that there is some positive dynamics at least in the integration of STEM in education [50,51,52].

## 6. Conclusions

This case study clarified the scope of CT and STEM philosophy in AET, documented perceptions of them, and finally revealed strengths and weaknesses of GAET. The research presented the value of CT and STEM in agriculture education and training, in context of the Agriculture 4.0 era.

Specifically, the final results of this survey (case study) are:

- There is a strong relationship between STEM philosophy and CT for teachers.
- There is a significant relationship between satisfaction with the school environment (IEKMC) and students’ opinion of the school environment.
- Educators consider STEM and STEM philosophy equally important for education.
- Students rate GAET higher than instructors.

The results of this research will be applied in the improvement of understanding CT and STEM philosophy in GAET. There is great interest in the implementation of CT and STEM in the EU by students and educators [40,47]. Finally, this research (case study) is a primary effort to research the usefulness of CT and STEM concepts in AET. It is a relevant study in the field of agricultural education and training in Greece. As the research was limited to a small sample, it is suggested for future research that:

- the sample is expanded nationwide;
- mixed research methods with a combination of quantitative and qualitative approaches are adopted for better analysis of the findings.

## Figures and Tables

**Table 1:** Population of Agriculture IEK of Metamorfosis city (IEKMC, education year 2020–2021).

	IEK (Total)	Viticulture & Oenology Technician
Staff	84	29
Students	310	51

**Table 2:** Statistical profile of opinion of STEM (n = 22).

Opinion of STEM	Aver.
Emphasis on applying knowledge to real-world problems	4.55
Support of critical thinking	4.55
Assistance in problem-solving	4.55
Creativity amplifying	4.55
It is important for National Economic Development	4.55

**Table 3:** Statistical profile of opinion of CT (n = 22).

Opinion of CT	Aver.
Assistance in problem-solving	3.55
Useful for the utilization of Information Technology and Science	3.55
Linked to STEM training	2.09
It is distinguished for its interdisciplinary application	3.18

**Table 4:** Statistical profile of opinion of didactic methodology of problem solving (n = 22).

Opinion of CT	Aver.
Simulation	3.09
Typical experiments	3.91
Through theoretical analysis (and solving exercises on paper)	3.09

**Table 5:** Correlation matrix of factors (STEM, CT, ET).

Spearman’s Rho	Opinion of STEM (Totally)	Opinion of CT (Totally)	Training Personnel
Opinion of STEM (totally)		0.7280 ** 0.000	−0.184 0.412
Opinion of CT (totally)	0.728 ** 0.000		−0.153 0.498
Training Personnel	−0.184 0.412	−0.153 0.498	

Note: **. correlation is Significant at the 0.01 level (2-tailed).

**Table 6:** Statistical profile of school environment (SE, n = 24).

Opinion of CT	Aver.
I apply knowledge to real world problems	4.75
I can solve problems more easily	1.13
I am learning to use algorithms to solve problems	0.88
I can analyze a problem, break it down into parts to solve it	3.63
I can use templates or models to solve a problem at school or in everyday life	3.75
I can identify patterns or models in a lesson or in everyday life	3.00
I can evaluate a model or model that appears to me in a course (e.g., physics) or in everyday life	1.25

**Table 7:** Score of overall satisfaction of IEKMC (n = 24).

Factors	Score (max = 5.0)
Infrastructure	1.63
Curriculum	1.0
Educators	0.75
Didactic techniques	1.00

**Table 8:** Correlation matrix of factors (school environment, SE, overall satisfaction, SAT).

Spearman’s Rho	Opinion of School Environment (IEKMC) (Totally) [SE]	Overall Satisfaction of School Environment (IEKMC) (Totally) [SAT]
Opinion of School Environment (IEKMC)(totally) [SE]		0.478 * 0.018
Overall Satisfaction of School Environment (IEKMC) (totally) [SAT]	0.478 * 0.180	

Note: *. correlation is significant at the 0.05 level (2-tailed).

## Data Availability

The data presented in this study are available on request from the corresponding author.
